# Irisin protects against cerebral ischemia reperfusion injury in a SIRT3-dependent manner

**DOI:** 10.3389/fphar.2025.1558457

**Published:** 2025-04-01

**Authors:** Yushuang Cong, Ruichun Guo, Chenglong Li, Qi Li, Sihua Qi

**Affiliations:** ^1^ Department of Anesthesiology, The Fourth Affiliated Hospital of the Harbin Medical University, Harbin, China; ^2^ Department of Anesthesiology, Peking University People’s Hospital, Beijing, China

**Keywords:** irisin, cerebral ischemia-reperfusion injury, MCAO, sirt3, apoptosis, oxidative stress

## Abstract

**Background:**

Cerebral ischemia-reperfusion (CIR) injury critically impacts stroke prognosis, yet effective therapeutic strategies remain limited. Irisin, an exercise-induced myokine, exhibits neuroprotective effects against cerebral ischemia. SIRT3, a mitochondrial deacetylase, is similarly implicated in mitigating ischemia-reperfusion injury. Given that irisin exerts protection via AMPK/PGC-1α pathway activation and SIRT3 acts downstream of PGC-1α , we hypothesized that SIRT3 mediates irisin's neuroprotection in CIR injury.

**Methods:**

In vivo cerebral ischemia-reperfusion injury was modeled by inducing transient middle cerebral artery occlusion (MCAO) in mice, while in vitro CIR conditions were replicated using oxygen-glucose deprivation (OGD) in PC12 neuronal cultures. To elucidate the mechanistic role of SIRT3, targeted interventions were implemented: SIRT3 expression was silenced via transfection with small interfering RNA (siRNA), and its enzymatic activity was pharmacologically inhibited using 3-TYP, a selective SIRT3 inhibitor. Apoptotic were systematically evaluated through TUNEL staining, Western blot analysis of caspase-3, Bax and Bcl-2. Oxidative stress parameters, including malondialdehyde (MDA) levels and glutathione (GSH) content, were measured using colorimetric assays. Neurological function in mice was quantified using the modified Neurological Severity Score (mNSS).

**Results:**

Our results demonstrated that irisin mitigates apoptosis and oxidative stress by dose-dependently activating SIRT3 signaling. At the optimal dosage, irisin effectively restored SIRT3 expression levels, reduced neuronal damage, and improved neurological recovery in CIR injury models. Notably, the therapeutic effects of irisin were significantly attenuated by 3-TYP, a specific SIRT3 inhibitor. Further validation through in vitro experiments revealed that SIRT3 overexpression synergistically enhanced irisin-mediated protection against OGD-induced injury, whereas SIRT3 knockout substantially diminished its efficacy.

**Conclusion:**

Our data shown that irisin exerted a protective role in CIR injury, at least in part, through SIRT3 activation. This study establishes the irisin/SIRT3 as a novel therapeutic target for ischemic stroke, providing mechanistic insights for future interventions.

## Introduction

Ischemic stroke has emerged as a leading global cause of mortality and long-term disability (Ganga et al., 2024; Peng et al., 2022). While pharmacological thrombolysis and mechanical thrombectomy currently represent the gold-standard therapeutic interventions (Berge et al., 2021; Jovin et al., 2022), these revascularization strategies present a paradoxical clinical challenge. Notably, cerebral ischemia-reperfusion (CIR) injury, a counterproductive consequence of restored blood flow, significantly diminishes treatment efficacy (Ng et al., 2022; Ng et al., 2021). Current clinical data reveal that nearly 50% of eligible patients fail to achieve meaningful clinical improvement post-revascularization, a statistic that underscores the growing clinical burden of CIR complications (Ma et al., 2025; Gebrezgiabhier et al., 2021). The pathophysiology of CIR injury involves a complex cascade of interdependent mechanisms, with oxidative stress emerging as a critical amplifier of neural damage (Zhang et al., 2024; Chouchani et al., 2016). Apoptosis represents the terminal and potentially modifiable endpoint in this destructive sequence (Zhang et al., 2023b; Liu et al., 2019a). Targeted interventions addressing these mechanisms may enhance clinical outcomes by mitigating CIR injury severity.

Irisin, a polypeptide cleaved from fibronectin type III domain-containing protein 5, is released into circulation during exercise (Kim et al., 2025). Preclinical studies have demonstrated irisin’s critical role in mitigating oxidative damage and suppressing apoptotic pathways across multiple experimental models (Wen et al., 2025; Ren et al., 2022; Kohan-Ghadr et al., 2021). Emerging evidence confirms its protective efficacy against ischemia-reperfusion injury in various organ systems, including myocardial, hepatic, and renal tissues (Cui et al., 2024; Liu et al., 2023b; Bi et al., 2019). Under cerebral ischemia conditions, irisin reduces apoptotic cell death following ischemia-reperfusion injury by enhancing mitochondrial dynamics and suppressing reactive oxygen species (ROS) production through activation of the PI3K/Akt/mTOR signaling pathway (Liu et al., 2023a). Clinical observations suggest a potential correlation between circulating irisin levels and improved short-term functional outcomes following ischemic stroke (Calik et al., 2022; Di Raimondo et al., 2020; Wu et al., 2019). Notably, widespread irisin distributed in different brain regions, including the hippocampus, cortex, and hypothalamus (Liu et al., 2024; Dupuis et al., 2024). These collective findings position irisin as a promising therapeutic candidate for cerebral ischemia-reperfusion injury. However, the precise molecular mechanisms underlying irisin’s neuroprotective actions remain incompletely characterized, particularly regarding its therapeutic potential in cerebral ischemia-reperfusion injury.

SIRT3, the predominant mitochondrial deacetylase (Barcena et al., 2024; Gao et al., 2022; Jin et al., 2022), shares regulatory parallels with irisin. SIRT3 plays a crucial role in mediating the beneficial effects of exercise by acting as a key molecular transducer of exercise-induced metabolic adaptations and cellular protection. (Li et al., 2023; Zhou et al., 2022; Cheng et al., 2016). SIRT3 has been mechanistically linked to its antioxidative and antiapoptotic properties across multiple tissue types (Tran et al., 2025; Juszczak et al., 2024; Jin et al., 2023; Di Paola et al., 2022). Emerging evidence positions SIRT3 as a critical mediator of ischemia-reperfusion injury pathophysiology, with demonstrated neuroprotective effects in cerebral models (Wang et al., 2024; Kong et al., 2024; Shen et al., 2021; Chen et al., 2024a). Notably, while irisin exerts protection via AMPK/PGC-1α pathway activation (Luo et al., 2022), and SIRT3 operates downstream of PGC-1α to modulate oxidative stress and apoptotic cascades (Gu et al., 2023; Zhang et al., 2020; Luo et al., 2022), no research has yet explored irisin’s impact on SIRT3 in ischemic brain tissue. This gap highlights the need to study the irisin-SIRT3 relationship in CIR injury.

In this study, we posited that SIRT3 plays a mediating role in the neuroprotective effects of irisin in CIR injury by mitigating oxidative stress and inhibiting apoptosis. Our objectives were to elucidate the relationship between irisin and SIRT3 in the context of CIR injury and to determine whether SIRT3 is instrumental in the protective effects exhibited by irisin. These objectives may collectively establish a mechanistic foundation for novel therapeutic strategies leveraging exercise-mimetic interventions in stroke management.

## Materials and methods

### Mouse models

Male C57BL/6 mice aged 8–12 weeks were reared in an alternating day and night environment and allowed to forage freely. All animal care procedures during the experiments followed the National Institutes of Health guidelines for the care and use of experimental animals.

### Middle cerebral artery occlusion model

MCAO was induced in male mice (20–25 g) following established protocols (Zeng et al., 2021). Briefly, anesthesia was achieved via intraperitoneal injection of sodium pentobarbital (100 mg/kg). Cerebral blood flow was continuously monitored using a laser Doppler flowmeter through a left cranial window. Through meticulous microsurgical dissection, the left common carotid artery (CCA), internal carotid artery (ICA), and external carotid artery (ECA) were carefully exposed while preserving vascular and vagal integrity.

MCAO was implemented by occluding the left middle cerebral artery for 45 min using a silicon-coated monofilament, with successful ischemia confirmed by >70% reduction in cerebral blood flow (Wang et al., 2021). Reperfusion was initiated by filament withdrawal and maintained for 24 h. Sham-operated controls underwent identical procedures without arterial occlusion.

Immediately post-reperfusion, irisin (25, 50, or 100 μg/kg) or saline vehicle was administered via tail vein injection. All experimental protocols strictly adhered to the 3 R principles (Reduction, Refinement, Replacement), minimizing animal usage while ensuring statistical validity.

### Assessment of neurologic deficits

The modified Neurological Severity Score (mNSS) was used to assess the neurological function of mice (Behrouzifar et al., 2018). Three days before MCAO, the mice were evaluated daily according to the mNSS to observe their behavioral function and allow acclimatization. The mice were weighed 24 h after IR, and their neurological function was assessed once again.

### Measurement of the infarct size

Mice were sacrificed as per standard institutional procedures. Mouse brains were removed at 24 h after reperfusion, rinsed with surface blood, and immediately placed at −20°C for 15 min. Thereafter, the brains were sectioned, soaked in 2% triphenyl tetrazolium chloride (T8877, Sigma, St Louis, MO, United States) solution, and stained for 30 min at 37°C in the dark. The infarct areas were then measured using Image-Pro Plus 6.0 software (Media Cybernetics, Inc., Rockville, Maryland, United States), and the infarct volumes are presented as the percentage of the total volume of the slices (Zhou et al., 2021).

### Terminal deoxynucleotidyl transferase dUTP nick end labeling (TUNEL) assay

TUNEL staining was performed using a commercial kit (11684817910, Roche, South San Francisco, CA, United States) according to the manufacturer’s instructions. Paraffin sections were dewaxed, hydrated, and then labeled with the TUNEL reaction mixture. The sections were observed at high magnification using a microscope. Five non-overlapping regions were randomly selected from each section to count the positive cells, and the results are presented as the apoptotic rate (×100%).

### Cell culture

The PC12 cell line (Shanghai Institutes for Biological Sciences, China) was maintained in high-glucose Dulbecco’s Modified Eagle Medium (DMEM; 10013CVRC, Corning, United States) supplemented with 10% heat-inactivated fetal bovine serum (03U16001DC, EallBio, China), 100 U/mL penicillin, and 100 μg/mL streptomycin (0312001A, EallBio, China) under standard culture conditions (37°C, 5% CO_2_, 95% humidified air). Cells at 70%–80% confluence were harvested for experiments (Peng et al., 2017).

To establish the oxygen-glucose deprivation (OGD) model, PC12 cells were incubated in glucose-free DMEM under hypoxic conditions (5% CO_2_, 1% O_2_, 94% N_2_) at 37°C for 8 h, followed by 3 h reoxygenation (5% CO_2_, 21% O_2_, 74% N_2_) in complete DMEM, as previously described (Si et al., 2018). During reoxygenation, cells were treated with or without irisin for 2 h.

### SIRT3 knockout and overexpression in vitro

SIRT3 knockdown was achieved by transfecting PC12 cells with Sirt3-targeting siRNA (sc-61555, Santa Cruz Biotechnology, United States) using Lipofectamine™ 8,000 transfection reagent (C0533, Beyotime, China) according to established protocols (Si et al., 2018). For transfection, 2 nmol/L siRNA was complexed with 8 μL Lipofectamine™ 8,000 in 100 μL Opti-MEM serum-free medium, incubated for 8 h, and applied to 70% confluent cells in 6-well plates for 48 h.

For SIRT3 overexpression, cells were transfected with pcDNA3.1 plasmids (128,034, Addgene, United States) using 0.8 μg plasmid DNA combined with 2 μL Lipofectamine™ 8,000 in 50 μL Opti-MEM (Sommer, 2017). The DNA-lipid complexes (54 μL total) were incubated for 30 min at room temperature before treating cells in RPMI 1640 supplemented with 10% calf serum. After 8 h exposure, cells were maintained in fresh medium for an additional 48 h. Transfection efficiency was validated by quantitative RT-PCR analysis. Transfected cells were subsequently passaged for downstream experiments.

### Determination of SIRT3 activity

Commercial kits (BML-AK557-000, Enzo Life Sciences Inc., United States) were used to detect the lysyl deacetylase activity of SIRT3 according to the manufacturer’s instructions. SIRT3 activity was normalized to the protein samples (40 μg) and measured with the spectrophotometer. Protein samples (40 μg) were added with specific substrates and incubated at 37°C for 45 min. Then, developer was added to the samples and incubated for an additional 45 min. The excitation wavelength and emission wavelength were 360 nm and 460 nm respectively.

### Measurement of cell viability using the CCK8 assay

CCK8 kits (CP002, SAB, United States) were used to assess cell viability in accordance with the manufacturer’s instructions. Cells were plated at a density of 10^3^ cells/well in 96-well plates, and each cell plate was inoculated with three replicates of cell samples. After the indicated treatment, CCK8 reagents were added to the culture medium, and the plates were incubated for 3 h. The absorbance was measured at 450 nm, and the absorbance value of each cell sample was recorded for further evaluation.

### Western blot analysis

PC12 cultures or mouse tissues were collected and proteins were isolated. Western blot was performed through a series of processes, including denaturation, electrophoresis, transmembrane, and closure, followed by incubation with primary antibodies against BCL-2, BAX (AF6139, AF0120, Affinity, United States), caspase-3, superoxide dismutase-2 (SOD2) (WL04004, WL02506, WanleiBio, Shenyang, China), acetylated SOD2 (ac-SOD2) (ab137037, Abcam, United Kingdom), nuclear factor erythroid 2-related factor 2 (Nrf2) (ab31163, Abcam, United Kingdom), SIRT3 (5490S, Cell Signaling Technology, United States), or β-actin (BM0627, BOSTER, CA, United States). Next, the membranes were washed and incubated with horseradish peroxidase-conjugated secondary antibodies. Finally, an enhanced chemiluminescence system (GE Healthcare, United Kingdom) was used to visualize protein bands. Image Lab software was used to analyze relative protein expression.

### Flow cytometric apoptosis assay

Annexin V-FITC/propidium iodide (PI) staining kits (7Sea Biotech, Shanghai, China) were used to assess apoptosis. PC12 cells were plated evenly at a density of 3 × 10^5^ per well in six-well plates. Three replicates of cells in each group were inoculated and then treated with different treatment conditions. After 48 h, the cells were collected, washed, resuspended, and subsequently stained with 5 μL of annexin V-FITC for 20 min and 10 μL of PI for 5 min in the dark. Apoptosis was then detected using the Accuri C6 Flow Cytometer (BD Biosciences, United States).

### Flow cytometric reactive oxygen species (ROS) assay

ROS assay kits (S0033S, Beyotime, Nantong, China) were used to detect ROS levels in PC12 cells, which were cultured and treated under different treatment conditions. The cells were washed, centrifuged, and resuspended, and the supernatant was removed and incubated with 50 μM DCFH-DA working solution at 37°C for 30 min in the dark. The resulting fluorescent images were then observed using the Accuri C6 Flow Cytometer (BD Biosciences, United States).

### Measurement of malondialdehyde (MDA) and glutathione (GSH) levels

Commercial kits (WLA048, WLA105, WanleiBio, Shenyang, China) were used to detect MDA and GSH levels according to the manufacturer’s instructions. MDA absorbance was measured at 532 nm. The GSH content was directly proportional to the concentration of the absorbance at 412 nm.

### Statistical analyses

Statistical analyses were performed using the GraphPad Prism software version 6.0 (GraphPad Software, San Diego, CA, United States). The data are represented as the mean ± standard error of the mean. The differences between multiple groups were assessed using one-way ANOVA, and the Tukey test with Bonferroni correction were performed for multiple comparisons. Statistical significance was set at P < 0.05. *A priori* power analysis using G*Power 3.0 (α = 0.05, power = 80%, effect size = 1.5 based on preliminary TTC staining data) yielded required sample sizes of 18 animals per group. Final group sizes were further optimized through preliminary experiments and historical data from analogous models. Exact sample sizes per group are reported in respective figure legends.

## Results

### Irisin decreases neuronal injury and improves neurological function following CIR injury

**FIGURE 1:**
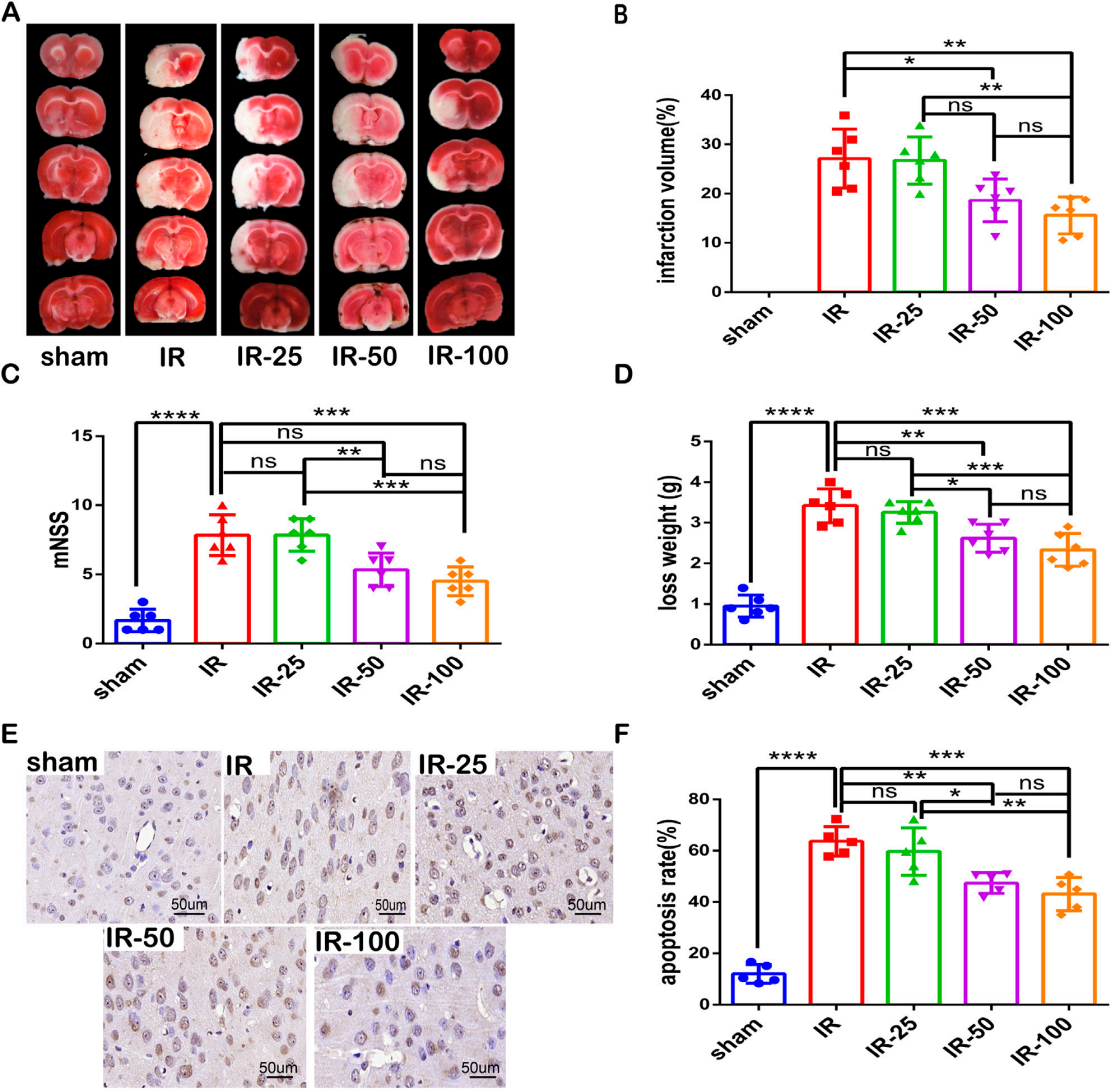
Irisin decreases neuronal injury and improves neurological function following CIR injury **(A)** TTC staining. **(B)** Infarct percentage of mouse brain tissues (n = 6). **(C)** The neurologic deficit scores (n = 6). **(D)** Weight loss (n = 6). **(E)** TUNEL assay of brain tissues. The black arrow points to cells with positive staining. Scale bar = 50 μm. **(F)** Apoptotic index (n = 3). IR: cerebral ischemia-reperfusion; IR-25: cerebral ischemia-reperfusion treated with irisin (25 μg/kg); IR-50: cerebral ischemia-reperfusion treated with irisin (50 μg/kg); IR-100: cerebral ischemia-reperfusion treated with irisin (100 μg/kg). Data are presented as the mean ± SEM. ^*^P < 0.05; ^**^P < 0.01; ^***^P < 0.001; ^****^P < 0.0001; ns, not significant.

To explore the optimum effective dose of irisin on CIR injury, different doses of irisin (25 μg/kg, 50 μg/kg, and 100 μg/kg) were administered to MCAO mice via the tail vein before reperfusion. After 24 h of reperfusion, we evaluated the infarct percentage of brain tissue, the behavioral function, weight loss and apoptotic ratio. As shown in Figures 1A, B, the infarction percentage of IR group was significantly higher than that of sham group, and irisin administered at 50 and 100 μg/kg doses apparently decreased the infarction percentage. The neurological deficit in IR group was more significant than that of sham group, and irisin treatment at 100 μg/kg dose reduced the neurological deficits (Figure 1C). Similarly, CIR-induced decline in the body weight in IR group was more significantly greater than that of sham group, and irisin treatment at 50 and 100 μg/kg doses reduced weight loss (Figure 1D). Moreover, the TUNEL assay showed that fewer apoptotic cells were present in mice after 24 h of reperfusion with an increase in the dose of irisin (Figures 1E, F). Based on our results, irisin 100 μg/kg was the optimum effective dose. These results indicated that following CIR injury, irisin administered via the tail vein decreased neuronal injury and improved neurological function in a dose-dependent manner.

### Irisin prevents SIRT3 expression and activity reduction, alleviates Oxidative stress and increases apoptotic protein levels induced by CIR injury

**FIGURE 2:**
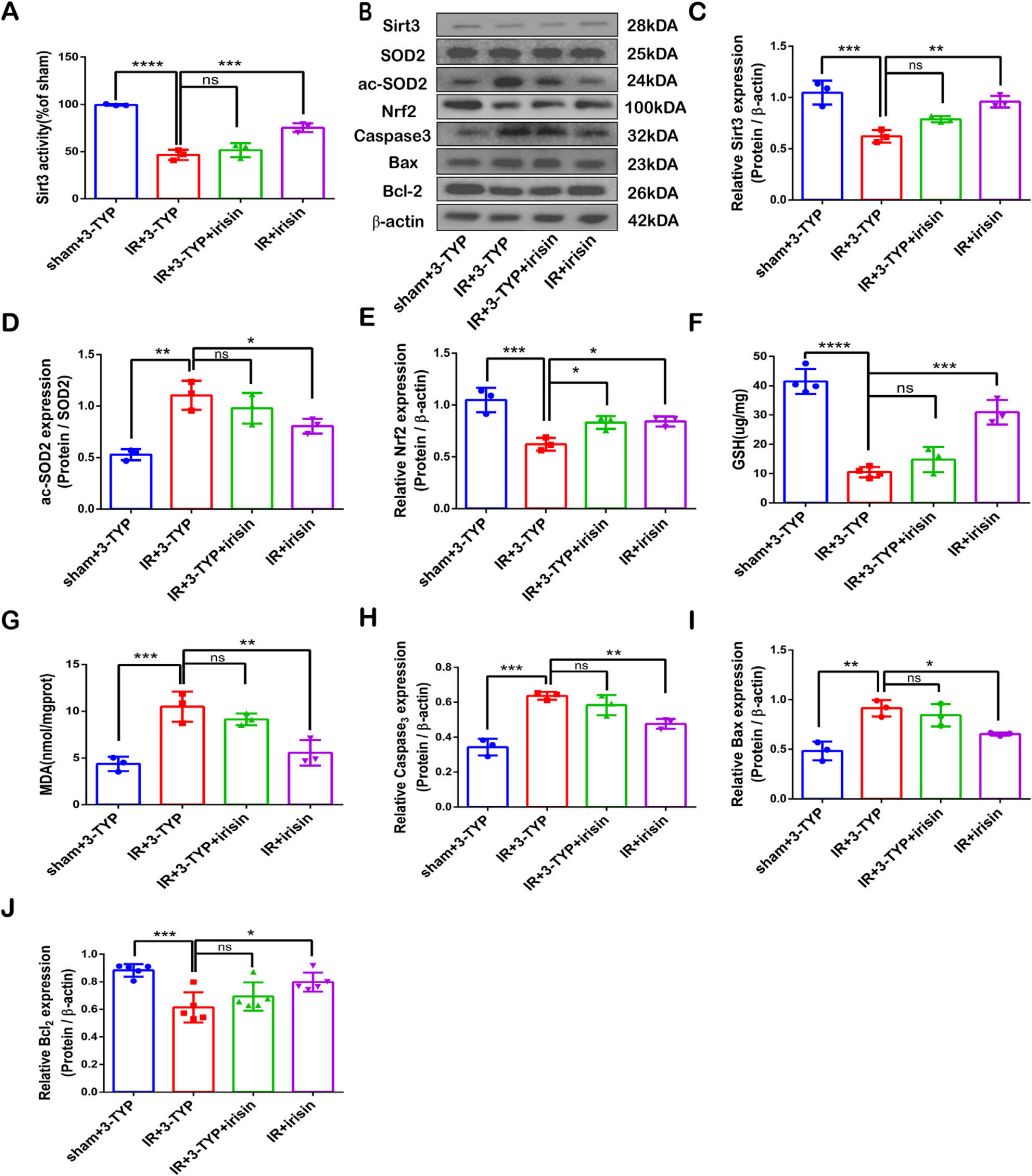
Irisin Prevents SIRT3 Expression and Activity Reduction, Alleviates Oxidative Stress and Increases Apoptotic Protein Levels Induced by CIR Injury **(A)** SIRT3 activity. **(B)** Representative protein images obtained by Western blot from different groups. **(C)** Expression of SIRT3 protein. **(D)** Expression of ac-SOD2 protein. **(E)** Expression of Nrf2 protein. **(F)** GSH content in brain tissues. **(G)** MDA content in brain tissues. **(H–J)** Expression of apoptosis-related proteins. Data are presented as the mean ± SEM, n = 3–6. P values were determined by ANOVA. ^*^P < 0.05; ^**^P < 0.01; ^***^P < 0.001; ^****^P < 0.0001; ns, not significant.

To explore the correlation between irisin and SIRT3 in CIR injury, we measured the expression and activity of SIRT3 at different doses of irisin after 24 h of reperfusion. Our results revealed that CIR led to SIRT3 expression and activity reduction, and these effects were reversed by irisin treatment at 50 and 100 μg/kg (Figures 2A–C). Irisin increased the expression and activity of SIRT3 in a dose-dependent manner. A recent study has shown that AdipoRon ameliorated chronic ethanol induced cardiac necroptosis via the SIRT3-SOD2-mtROS pathway (Qian et al., 2025). SOD2 is a primary target of SIRT3, and a most important indices for evaluating oxidative stress. Also, the expression of SOD2 and ac-SOD2 has often been used to demonstrate the activity of SIRT3. We measured the expression of SOD2 and ac-SOD2. Our results revealed that CIR led to SOD2 hyperacetylation, and this effect was reversed by irisin treatment at 50 and 100 μg/kg (Figure 2D). Previous data have shown that irisin plays a pivotal role in reducing oxidative stress and alleviating apoptosis via uncoupling protein-2 (UCP2) in pulmonary IR injury (Chen et al., 2017). To explore the protective mechanism of irisin on CIR injury, we measured the levels of MDA and GSH as well as the expression of Nrf2 and apoptosis-associated proteins induced by CIR injury after different doses of irisin treatment. Irisin treatment at 50 μg/kg and 100 μg/kg increased Nrf2 expression compared with that in the IR group (Figure 2E). Following CIR injury, MDA levels were significantly elevated and GSH levels were reduced; these effects were reversed by irisin treatment in a dose-dependent manner (Figures 2F, G). Irisin treatment at 100 μg/kg reduced caspase-3 and BAX protein expression and increased BCL-2 expression compared with those in the IR group (Figure 2H−J). These results suggest that irisin could reduce oxidative stress, alleviate apoptosis, and exert its protective effect in CIR injury in a dose-dependent manner.

### Irisin protects PC12 cells exposed to OGD against neuronal injury

**FIGURE 3:**
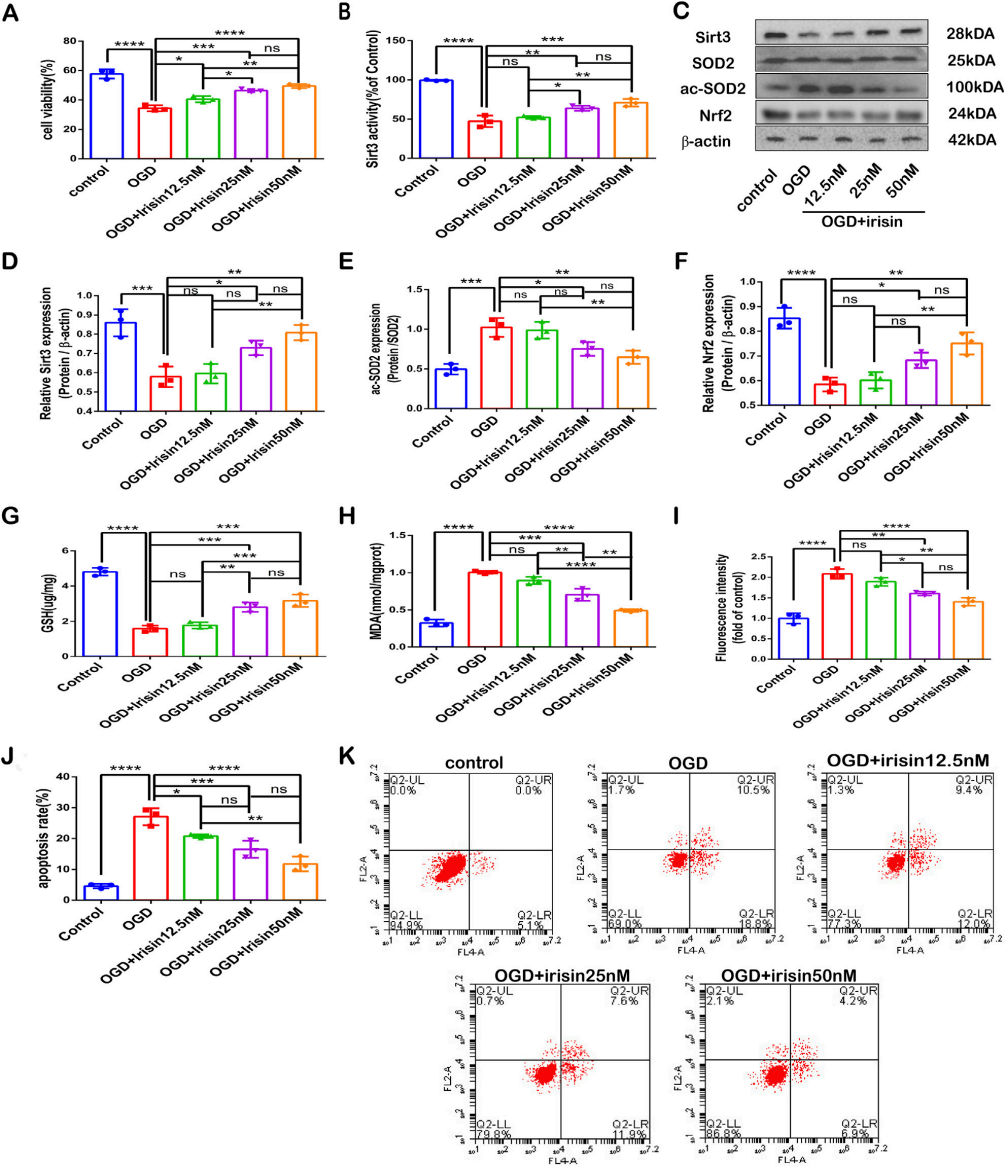
Irisin protects PC12 cells exposed to OGD against neuronal injury **(A)** Cell death. **(B)** SIRT3 activity. **(C)** Representative protein images obtained by Western blot from different groups. **(D)** Expression of SIRT3. **(E)** Expression of ac-SOD2. **(F)** Expression of Nrf2 protein. **(G)** Cellular GSH content. **(H)** Cellular MDA content. **(I)** Flow cytometry analysis to assess intracellular ROS. **(J)** Cell apoptosis presented as the apoptotic index (×100%). **(K)** Flow cytometry analysis to assess cell apoptosis. Data are presented as the mean ± SEM, n = 3. OGD: PC12 cells subjected to oxygen-glucose deprivation; irisin12.5 nM: PC12 cells treated with irisin (12.5 nM); irisin 25 nM: PC12 cells s treated with irisin (25 nM); irisin 50 nM: PC12 cells treated with irisin (50 nM). P values were determined by ANOVA. ^*^P < 0.05; ^**^P < 0.01; ^***^P < 0.001; ^****^P < 0.0001; ns, not significant.

To further verify the neuroprotective effects of irisin, PC12 cells were exposed to OGD *in vitro*. Irisin significantly enhanced cell proliferation and viability at 8 h in a dose-dependent manner (Figure 3A). An increased therapeutic dose of irisin also attenuated SIRT3 expression and activity reduction and SOD2 hyperacetylation, increased Nrf2 expression (Figure 3B−F). Treatment with an increased therapeutic dose of irisin reduced ROS and MDA production and enhanced the levels of GSH induced by OGD (Figure 3G−I). and reduced the apoptosis rate, as demonstrated by flow cytometric apoptosis assay (Figures 3J, K). These data suggest that irisin exerts its protective effects by alleviating apoptosis and reducing oxidative stress. Furthermore, irisin increases the expression and activity of SIRT3 in a dose-dependent manner.

### SIRT3 participates in the protective effects of irisin against OGD injury

**FIGURE 4:**
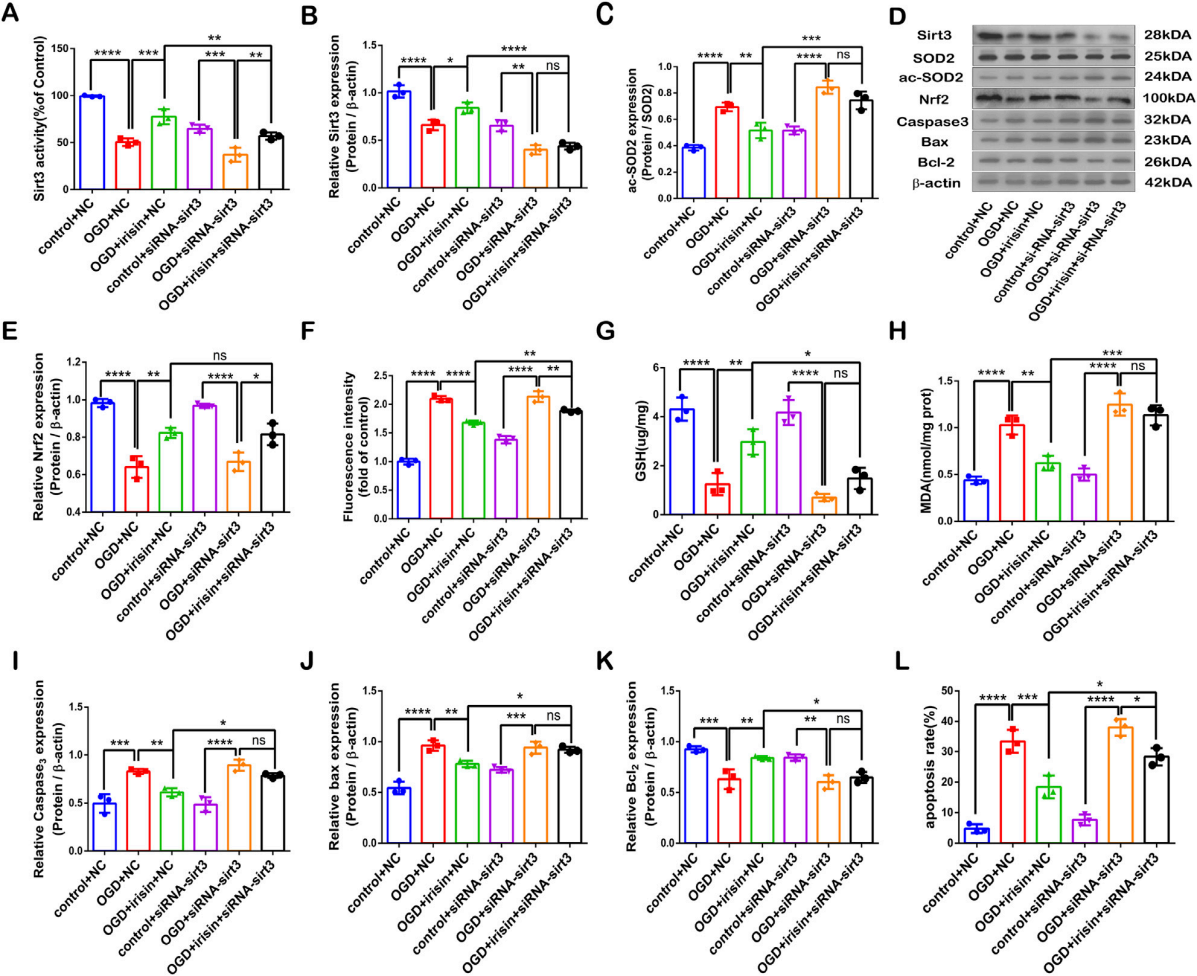
SIRT3 participates in the protective effects of irisin against OGD injury. Modulation of the protective effects of irisin in PC12 cells transfected with *Sirt3* siRNA or non-targeting siRNA and exposed to OGD with or without irisin pretreatment **(A)** SIRT3 activity. **(B)** Expression of SIRT3. **(C)** Expression of ac-SOD2. **(D)** Representative protein images obtained by Western blot from different groups. **(E)** Expression of Nrf2 protein. **(F)** Intracellular ROS levels examined by DCFH-DA fluorescence. **(G)** Cellular GSH content. **(H)** Cellular MDA content. **(I–K)** Expression of apoptosis-related proteins. **(L)** Cell apoptosis presented as the apoptotic index (×100%). Data are presented as the mean ± SEM, n = 3. NC: PC12 cells transfected with scrambled-sequence siRNA, serving as the negative control; siRNA-sirt3: PC12 cells with SIRT3 knockdown using SIRT3-targeting siRNA. P values were determined by ANOVA. ^*^P < 0.05; ^**^P < 0.01; ^***^P < 0.001; ^****^P < 0.0001; ns, not significant.

**FIGURE 5:**
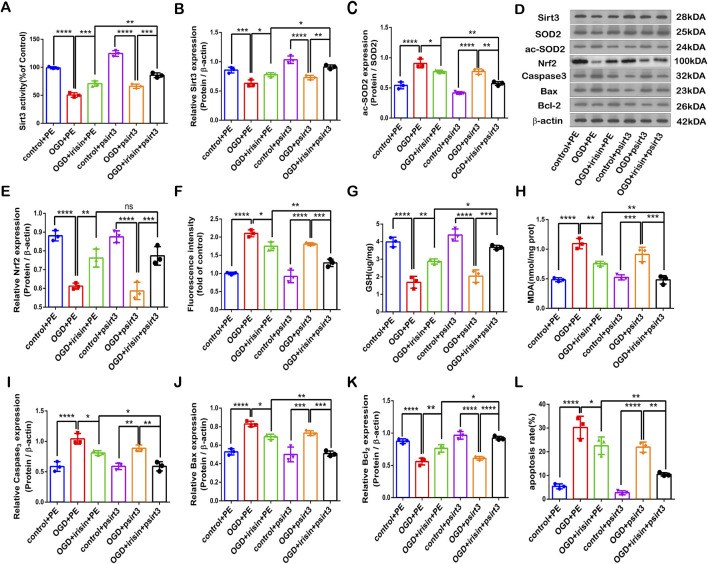
SIRT3 participates in the protective effects of irisin against OGD injury. Modulation of the protective effects of irisin in PC12 cells transfected with a plasmid carrying *Sirt3* gene (psirt3) or empty plasmid (PE) and exposed to OGD with or without irisin pretreatment **(A)** SIRT3 activity. **(B)** Expression of SIRT3. **(C)** Expression of ac-SOD2. **(D)** Representative protein images obtained by Western blot from different groups. **(E)** Expression of Nrf2 protein. **(F)** Intracellular ROS levels examined by DCFH-DA fluorescence. **(G)** Cellular GSH content. **(H)** Cellular MDA content. **(I–K)** Expression of apoptosis-related proteins. **(L)** Cell apoptosis presented as the apoptotic index (×100%). Data are presented as the mean ± SEM, n = 3. PE: PC12 cells transfected with the empty pcDNA3.1 vector, serving as the negative control; psirt3: PC12 cells overexpressing SIRT3 via transfection with a pcDNA3.1 plasmid encoding a Flag-tagged SIRT3 sequence. P values were determined by ANOVA. ^*^P < 0.05, ^**^P < 0.01, ^***^P < 0.001, ^****^P < 0.0001.

To investigate whether SIRT3 is involved in the protective effects of irisin during OGD injury, SIRT3 was knocked down by siRNA transfection in PC12 cells. The SIRT3 inhibitory effect is shown in Figures 4A, B. *Sirt3-targetting* siRNA inhibited SIRT3 expression and activity. Our results showed that the effects of irisin treatment were weakened after the inhibition of SIRT3 during OGD injury (Figure 4C−L). To further assess whether SIRT3 is involved in the protective effects of irisin, SIRT3 was overexpressed in PC12 cells. The effect of SIRT3 overexpression is shown in Supplementary Figure 5A, B. SIRT3 overexpression increased the production of SIRT3 at both the transcription and translation levels. SIRT3 overexpression enhanced the effects of irisin treatment (Figure 5C−L). SIRT3 knockdown inhibited, while its overexpression promoted, the irisin-mediated enhancement of cell proliferation. These data suggest that irisin exerts protective effects against OGD injury via SIRT3.

### SIRT3 participates in the neuroprotective effect of irisin against CIR injury

**FIGURE 6:**
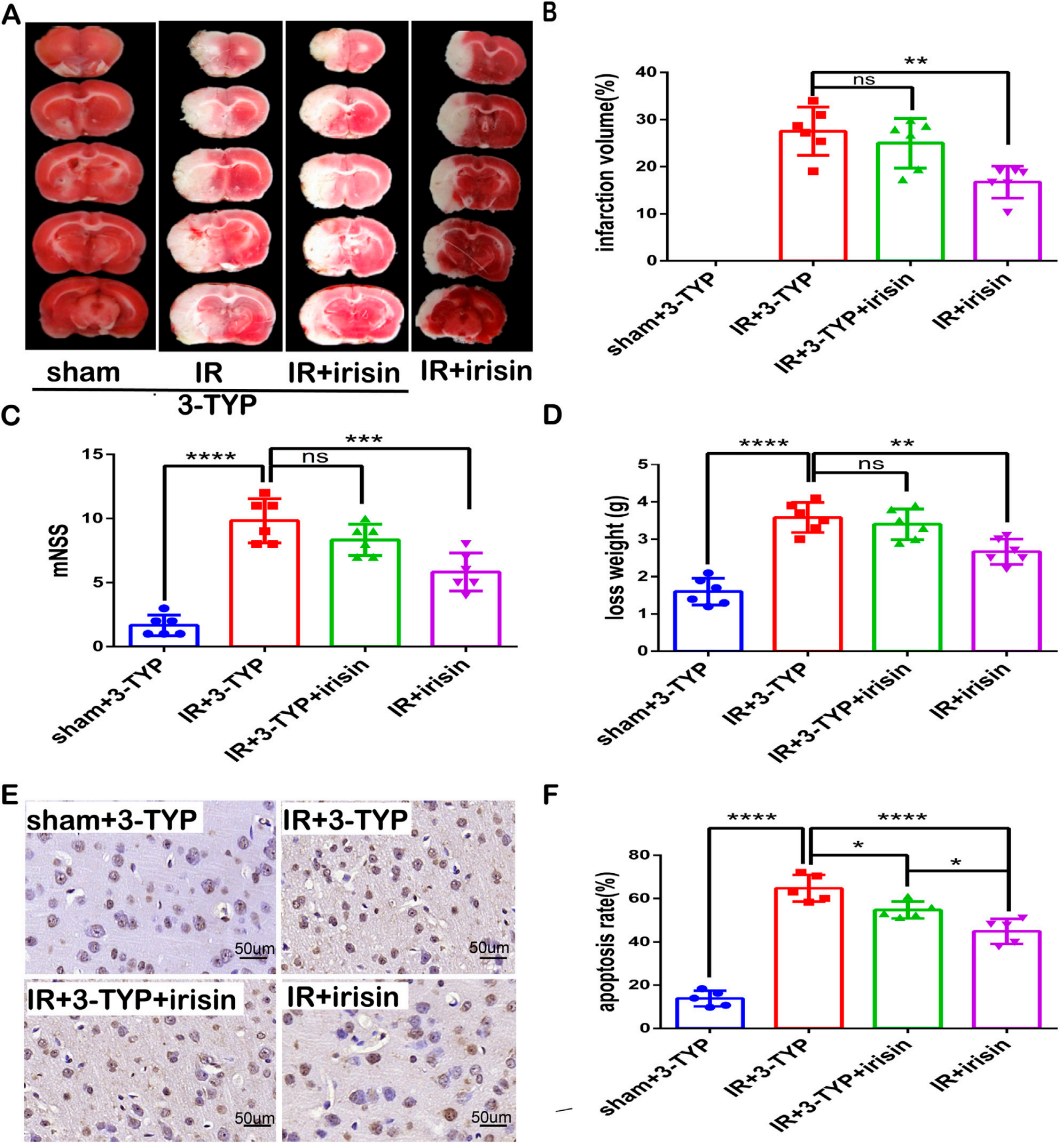
SIRT3 participates in neuroprotection of irisin against CIR injury **(A)** TTC staining. **(B)** Infarct percentage of mouse brain tissues (n = 6). **(C)** The neurologic deficit scores (n = 6). **(D)** Weight loss (n = 6). **(E)** TUNEL assay of brain tissues. The black arrow points to cells with positive staining. Scale bar = 50 µm. **(F)** Apoptosis presented as the apoptotic index (n = 3). 3-TYP: a SIRT3 inhibitor. Data are presented as the mean ± SEM. ^*^P < 0.05; ^**^P < 0.01; ^***^P < 0.001; ^****^P < 0.0001; ns, not significant.

To confirm whether SIRT3 participates in the neuroprotective effect of irisin against CIR injury, 3-TYP, a SIRT3 inhibitor that has little influence on the mouse brain (Lu et al., 2020), was used to achieve the inhibition of SIRT3 expression and activity. We found that the inhibition of SIRT3 nullified the neuroprotection of irisin by increasing infarct volume percentage, the neurological deficit scores, and weight loss (Figures 6A–D). The TUNEL assay revealed that fewer apoptotic cells were both observed in the IR + irisin group and in the IR+3-TYP + irisin group compared to the IR+3-TYP group, when fewer apoptotic cells were observed in IR + irisin group than that in the IR+3-TYP + irisin group (Figures 6E, F). These findings indicate that SIRT3 plays an important role in the neuroprotective effects of irisin against CIR injury, at least in part.

### SIRT3 participates in irisin-mediated decrease in apoptosis and oxidative stress induced by CIR injury

**FIGURE 7:**
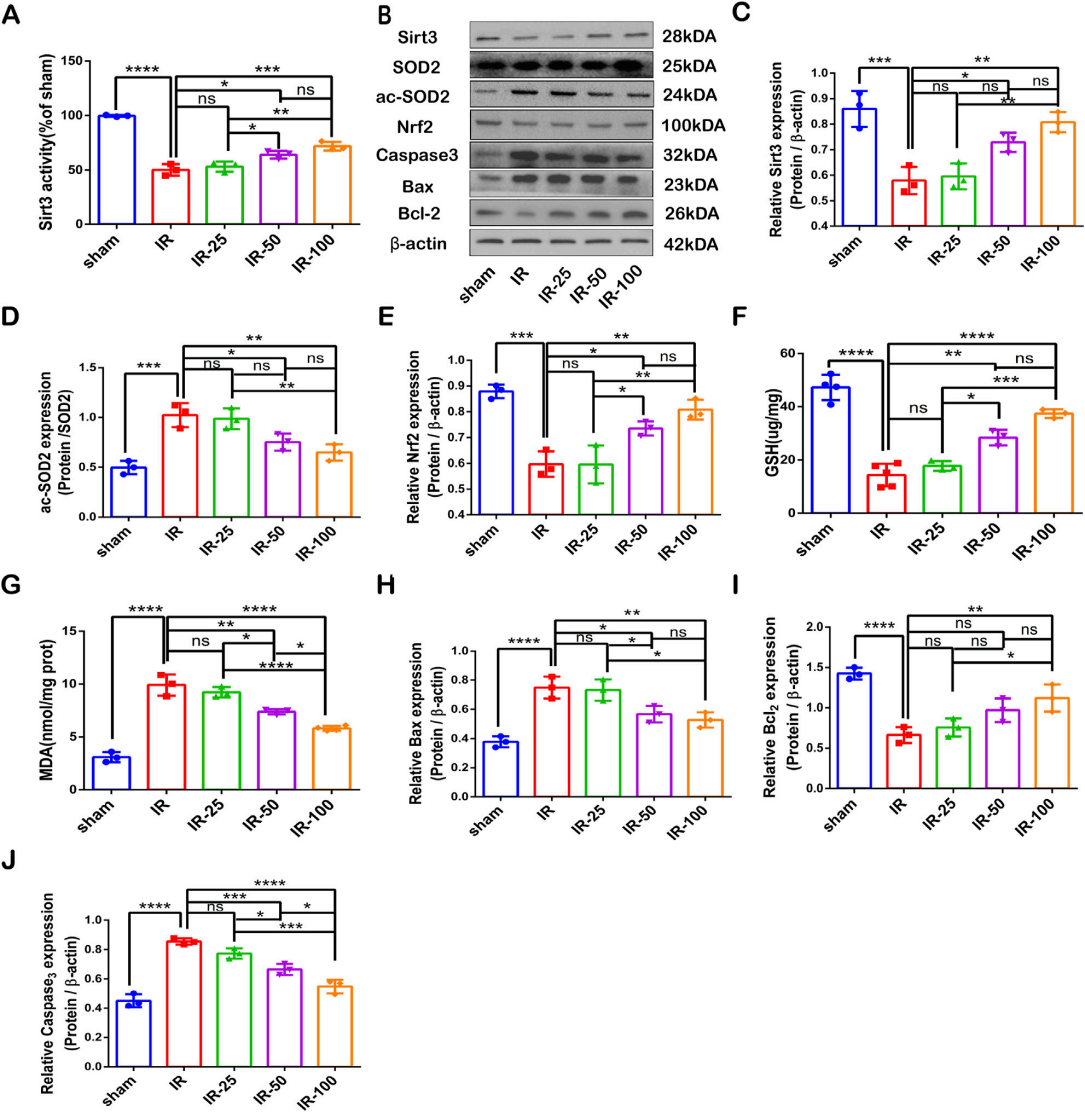
SIRT3 participates in the irisin-mediated decrease in apoptosis and oxidative stress induced by CIR injury **(A)** SIRT3 activity. **(B)** Representative protein images obtained by Western blot from different groups. **(C)** Expression of SIRT3 protein. **(D)** Expression of ac-SOD2 protein. **(E)** Expression of Nrf2 protein. **(F)** GSH content in brain tissues. **(G)** MDA content in brain tissues. **(H–J)** Expression of apoptosis-related proteins. Data are presented as the mean ± SEM, n = 3–6. P values were determined by ANOVA. ^*^P < 0.05; ^**^P < 0.01; ^***^P < 0.001; ^****^P < 0.0001; ns, not significant.

Our results revealed that irisin could not rescue SIRT3 expression reduction and activity induced by 3-TYP during CIR injury (Figures 7A–C). We also found that irisin treatment increased Nrf2 and GSH levels as well as reduced SOD2 hyperacetylation and MDA levels, and then the inhibition of SIRT3 reversed the protective effect of irisin (Figures 7D–G). Furthermore, irisin treatment reduced caspase-3 and BAX protein expression and increased BCL-2 expression, the effect of which was nullified by the inhibition of SIRT3 (Figure 7H−J). These data suggest that irisin exerts neuroprotective effects against CIR-induced apoptosis and oxidative stress through SIRT3.

## Discussion

Our results demonstrated that irisin mitigates apoptosis and oxidative stress by dose-dependently activating SIRT3 signaling. At the optimal dosage, irisin effectively restored SIRT3 expression levels, reduced neuronal damage, and improved neurological recovery in CIR injury models. Notably, the therapeutic effects of irisin were significantly attenuated by 3-TYP, a specific SIRT3 inhibitor. Further validation through *in vitro* experiments revealed that SIRT3 overexpression synergistically enhanced irisin-mediated protection against OGD-induced injury, whereas SIRT3 knockout substantially diminished its efficacy.

Despite significant advancements in ischemic stroke management, pharmacological thrombolysis and mechanical thrombectomy remain the only clinically approved interventions and the most effective reperfusion strategies (Berge et al., 2021). However, vascular recanalization may paradoxically exacerbate CIR injury (Ng et al., 2021). CIR injury encompasses a dynamic and multifaceted pathophysiological cascade characterized by calcium overload, oxidative stress, inflammatory activation, intracellular acidosis, mitochondrial dysfunction (Zhou et al., 2018), and programmed cell death (Zhu et al., 2018; Zhang et al., 2023b). Consequently, targeting these pathological mechanisms represents a promising neuroprotective approach (Catanese et al., 2017). Notably, oxidative stress has been identified as a critical driver of CIR-related damage (Li et al., 2021; Zhang et al., 2024), with apoptosis serving as the terminal yet potentially modifiable stage in this pathological continuum (Wang et al., 2021). Based on this mechanistic framework, our study specifically investigated the therapeutic potential of irisin in mitigating oxidative stress and apoptosis during CIR injury.

Since its discovery in 2012, Irisin, a polypeptide cleaved from fibronectin type III domain-containing protein 5, is released into circulation during exercise (Kumar et al., 2025; Bostrom et al., 2012). Accumulating evidence highlights its pivotal roles in mitigating oxidative stress and attenuating apoptosis (Wen et al., 2025; Alshanqiti et al., 2023). For instance, irisin has been shown to protect against cardiac ischemia-reperfusion (IR) injury by suppressing suppressing oxidative stress and cardiomyocyte apoptosis (Lu et al., 2020). Similarly, in lung IR injury models, irisin ameliorates alveolar epithelial cell apoptosis via upregulation of uncoupling protein 2 (UCP2) (Chen et al., 2017). Furthermore, its ability to inhibit oxidative stress has been independently validated in neuronal systems (Sadier et al., 2024). Consistent with these findings, our results showed that the optimal dose of irisin reduced oxidative stress by upregulating GSH, decreasing MDA levels and increasing Nrf2 protein expression in brain tissues after CIR injury. Concurrently, the optimal dose of irisin also reduced the cell apoptosis index, as observed by TUNEL staining, and upregulated BCL-2 levels while decreasing caspase-3 and BAX protein levels at 24 h after reperfusion. These neuroprotective effects were further corroborated by *in vitro* experiments. Although tissue-specific signaling pathways may underlie irisin’s actions, its consistent anti-oxidative and anti-apoptotic efficacy across multiple organs strongly supports its translational potential for clinical IR injury management.

Emerging evidence demonstrates that irisin exerts protective effects across multiple disease models by modulating sirtuin signaling pathways, particularly SIRT3(Chi et al., 2022; Sadier et al., 2024; Zhao et al., 2025). Notably, exercise—a physiological inducer of irisin—upregulates SIRT3 expression, which has been well-documented to mitigate oxidative stress and apoptosis in CIR injury (Cai et al., 2021; Chen et al., 2024b; Xiaowei et al., 2023). For instance, Liu et al. (2020) reported that luteolin exacerbated CIR damage through SIRT3 inhibition, whereas Liu et al. (2019b) demonstrated melatonin-enhanced neuroprotection via SIRT3 activation. Intriguingly, irisin exerts protection via AMPK/PGC-1α pathway activation (Luo et al., 2022), and SIRT3 operates downstream of PGC-1α to modulate oxidative stress and apoptotic cascades (Gu et al., 2023). Notably, CIR injury significantly suppressed both PGC-1α and SIRT3 expression, whereas irisin treatment concomitantly rescued their levels, as demonstrated in our prior study. Based on this mechanistic cascade, we propose a novel paradigm: SIRT3 serves as the central mediator of irisin’s neuroprotection against CIR injury, primarily through dual modulation of oxidative stress and apoptotic pathways. To our knowledge, this study is the first to establish the irisin/SIRT3 axis as a therapeutic target for CIR injury management.

To delineate the role of SIRT3 in irisin-mediated neuroprotection against cerebral ischemia-reperfusion (CIR) injury, we systematically evaluated SIRT3 expression and activity in mouse brain tissues across escalating irisin doses post-CIR. Our data revealed that CIR injury markedly suppressed both SIRT3 protein levels and deacetylase activity. Strikingly, irisin administration dose-dependently reversed these deficits. This dose-response relationship was further validated *in vitro*, where irisin enhanced SIRT3 expression and activity in OGD-challenged PC12 cells. Crucially, the irisin-induced SIRT3 upregulation paralleled its neuroprotective efficacy against both IR- and OGD-induced oxidative stress and apoptosis, strongly implicating SIRT3 as a central mechanistic node in irisin’s therapeutic action.

To definitively establish SIRT3’s essential role in irisin-mediated neuroprotection against CIR injury, we employed complementary genetic and pharmacological approaches in MCAO and OGD models: PC12 cells were subjected to SIRT3 knockdown via siRNA or overexpression through plasmid transfection.3-TYP, a specific SIRT3 inhibitor, was administered during CIR challenge. Exogenous irisin significantly attenuated OGD-induced oxidative stress and apoptosis. siRNA-mediated SIRT3 knockdown abolished irisin’s antioxidative effect and its anti-apoptotic capacity. SIRT3 overexpression synergistically enhanced irisin’s protection. Co-treatment with 3-TYP attenuated irisin’s neuroprotection, further confirming SIRT3’s mechanistic centrality. This multi-modal verification conclusively positions SIRT3 as the indispensable mediator of irisin’s therapeutic effects against ischemic brain injury.

Our study revealed that CIR injury significantly increased ac-SOD2 levels, indicative of impaired mitochondrial antioxidant defense. Notably, irisin administration dose-dependently rescued SIRT3 expression and normalized SOD2 hyperacetylation, paralleling its neuroprotective effects on apoptosis. This mechanistic interplay aligns with reported SIRT3-SOD2 axis activation in other protective paradigms: Melatonin ameliorates diabetic cerebral IR injury via Akt-SIRT3-SOD2-mediated mitochondrial stabilization (Liu et al., 2021); Quercetin attenuates cardiomyocyte apoptosis through Sirt3/SOD2-dependent ROS suppression (Xiong et al., 2024). Crucially, SOD2 is the primary mitochondrial antioxidant enzyme directly deacetylated and activated by SIRT3(Cheng et al., 2017; Zhou et al., 2014), while other SIRT3 targets mainly regulate metabolism rather than oxidative stress (Jia et al., 2024; Zhang et al., 2023a). decreased SOD2 activity is the earliest event in the mitochondrial ROS burst (Miao and St Clair, 2009; Jiang et al., 2021). SOD2 gene polymorphism was significantly associated with the prognosis of ischemic stroke (Yang et al., 2021). Collectively, these findings delineate a SIRT3/SOD2 signaling axis as the mechanistic cornerstone of irisin’s neuroprotection against CIR injury.

Our study reveals an intriguing paradox in SIRT3’s role as a mediator of irisin’s neuroprotective effects against CIR injury. SIRT3 inhibition using 3-TYP abolished irisin-conferred neuroprotection, as evidenced by exacerbated infarct volume, neurological deficits, and body weight loss. Paradoxically, TUNEL assay results demonstrated significantly reduced apoptosis in the IR+3-TYP + irisin group compared to the IR+3-TYP group. This apparent discrepancy may arise from two non-exclusive mechanisms: 3-TYP -mediated SIRT3 knockdown but do not fully eliminate SIRT3 activity, allowing residual SIRT3 to mediate irisin’s partial anti-apoptotic effects. Irisin may engage alternative cytoprotective mechanisms independent of SIRT3, such as direct modulation of apoptotic effectors. Notably, while CIR injury coordinately suppressed both Nrf2 and SIRT3 expression, irisin treatment restored their levels through independent regulatory mechanisms, as evidenced by the preserved Nrf2 upregulation following SIRT3 genetic perturbation. This observation strongly supports the existence of parallel neuroprotective pathways—one involving SIRT3-mediated oxidative stress mitigation and another relying on Nrf2-driven antioxidant responses. However, the precise crosstalk between these pathways remains to be elucidated and constitutes a key focus of our ongoing research.

Several limitations warrant consideration in interpreting our findings: First, while this study establishes SIRT3 as a critical mediator of irisin’s anti-apoptotic and antioxidant effects, the upstream regulatory network governing SIRT3 activation remains incompletely characterized. Systematic interrogation of upstream signaling hubs and post-translational modifications modulating SIRT3 activity will be essential to map the full mechanistic landscape. Second, our experimental paradigm employed acute exogenous irisin administration in mice. To bridge translational gaps, future investigations should prioritize exercise-mimetic interventions to enhance endogenous irisin production, thereby better recapitulating physiological neuroprotection in clinical settings.

In summary, our findings delineate a SIRT3-centric mechanism through which irisin confers neuroprotection against CIR injury by coordinated mitigation of oxidative stress and apoptotic cascades. While SIRT3-dependent pathways account for a substantial portion of irisin’s therapeutic efficacy, the preserved Nrf2 activation in SIRT3-compromised models suggests auxiliary cytoprotective mechanisms warranting further exploration. These objectives may collectively establish a mechanistic foundation for novel therapeutic strategies leveraging exercise-mimetic interventions in stroke management.

## Data Availability

The datasets presented in this study can be found in online repositories. The names of the repository/repositories and accession number(s) can be found below: https://www.jianguoyun.com/p/DXzvpVgQtpOYDRjh4OcFIAA.
